# Fasciclin-like arabinogalactan protein gene expression is associated with yield of flour in the milling of wheat

**DOI:** 10.1038/s41598-017-12845-y

**Published:** 2017-10-02

**Authors:** Ravi C. Nirmal, Agnelo Furtado, Parimalan Rangan, Robert J. Henry

**Affiliations:** 1 Queensland Alliance for Agriculture and Food Innovation, University of Queensland, Brisbane, St Lucia, Qld, Australia; 2 Division of Genomic Resources, ICAR-National Bureau of Plant Genetic Resources, New Delhi, 110012 India

## Abstract

A large portion of the global wheat crop is milled to produce flour for use in the production of foods such as bread. Pressure to increase food supplies sustainably can be address directly by reducing post-harvest losses during processes such as flour milling. The recovery of flour in the milling of wheat is genetically determined but difficult to assess in wheat breeding due to the requirement for a large sample. Here we report the discovery that human selection for altered expression of putative cell adhesion proteins is associated with wheats that give high yields of flour on milling. Genes encoding fasciclin-like arabinogalactan proteins are expressed at low levels in high milling wheat genotypes at mid grain development. Thirty worldwide wheat genotypes were grouped into good and poor millers based flour yield obtained from laboratory scale milling of mature seeds. Differentially expressed genes were identified by comparing transcript profiles at 14 and 30 days post anthesis obtained from RNA-seq data of all the genotypes. Direct selection for genotypes with appropriate expression of these genes will greatly accelerate wheat breeding and ensure high recoveries of flour from wheat by resulting in grains that break up more easily on milling.

## Introduction

Wheat is the leading crop in the temperate world^1^. It provides 20% of the total calories and proteins consumed worldwide^2^. Wheat is ground to flour by the process of dry milling to make various food products such as bread which are suitable for human consumption. From a milling perspective wheat grain has three main components; endosperm, bran and germ, each with different mechanical properties^3^. In milling, the endosperm of the grain is scraped from the bran by passing the grain between metal rollers and sieving out the flour derived from the small particles of endosperm released. Clean separation of endosperm from bran and germ is desired in this process^4^. The efficiency of the process as measured by the yield of flour is the subject of selection by wheat breeders developing new wheat varieties for these products. The recovery of flour is largely determined by genotype and milling process with recoveries usually in the range 70 to 80%^5^. However, the maximum theoretical yield expected is around 85% which is the percentage of endosperm in the wheat grain^4^. In wheat breeding, small scale laboratory test mills are widely used for the selection of wheat genotypes that deliver a higher flour yield. However, this process is difficult because the test has poor repeatability and still requires more than 1 kg of grain. These quantities of grain are not available from single plants to allow early generation selection for this key trait.

Flour yield is a complex trait controlled by interaction between genotype, environment and processing^5–8^. Three main factors which influence milling performance of the wheat are grain hardness^9^, endosperm to bran ratio and ease of separation of endosperm from bran^3^. Soft wheats usually produce ~2% less flour than the hard wheats due to differences in mechanical properties of the grains^4^. Several QTLs associated with milling yield have been reported, on chromosome 6A^10^, 3B^11^, 1DL, 2 AS, 2BS, 2DS, 3DL, 4AC, 4DS, 5AL, 6 AC, 6DS^12^, 1B, 2A, 2B, 3B, 6A, 7 A, 7B^13^. Co-relation between starch granule size distribution (SGSD) and milling yield has been demonstrated by Edwards *et al*.^14^ in hard wheats. It was suggested that an even size distribution (increased volume of B- and C- type small granules and reduced % volume of A-type large granules) of starch granules is genetically controlled and associated with higher flour yield. Edwards *et al*.^9^ showed that 68% of the variation in hard wheats (Pina-D1a/Pinb-D1b) could be explained by combined effect of grain hardness and SGSD. Endosperm strength and stiffness measured by SKCS has also been shown to correlate with milling yield^15^. Much of this may explain environmental rather than genetic variation in milling performance. Despite of all this analysis the genetic and environmental components of variation and their molecular basis remain unclear.

Genetics controls a large part of the variation in flour yield. For example, Laidig *et al*.^16^ (2017) reported that genotype was a large contributor to variation in yield of wheat in Germany (1983–2014). Knowledge of the molecular basis^17^ of the intense human selection for high flour yield in breeding modern wheat genotypes would greatly accelerate the breeding of wheat combining high productivity and the grain quality required by consumers. This capability will be of great value in rapidly adapting wheat to changing and variable climates. The main objective of this study was to identify candidate genes which may control flour yield of wheat. RNA Seq of 30 diverse worldwide wheat genotypes was conducted at 14 and 30 days post anthesis (DPA) and gene expression was analysed relative to flour yield obtained on laboratory milling of the mature grain.

## Results

### Milling yield

Milling yield of the thirty wheat genotypes measured as % of total flour yield is shown in Table 1. Among the thirty genotypes, 17 were identified as high milling wheat genotypes (HM-group) (>/ = 77%) and 13 were identified as poor milling wheat genotypes (PM-group) (<77%). The milling yield of genotype NW51A was the lowest (71.4%) while that of Ellison was the highest (79.7%), respectively (Table 1). The milling yield of Gregory and Bobwhite could not be determined due to lack of grain samples from these trails. However, these genotypes are commercially known as high milling wheats and thus were placed into the HM-group.

**Table 1:**
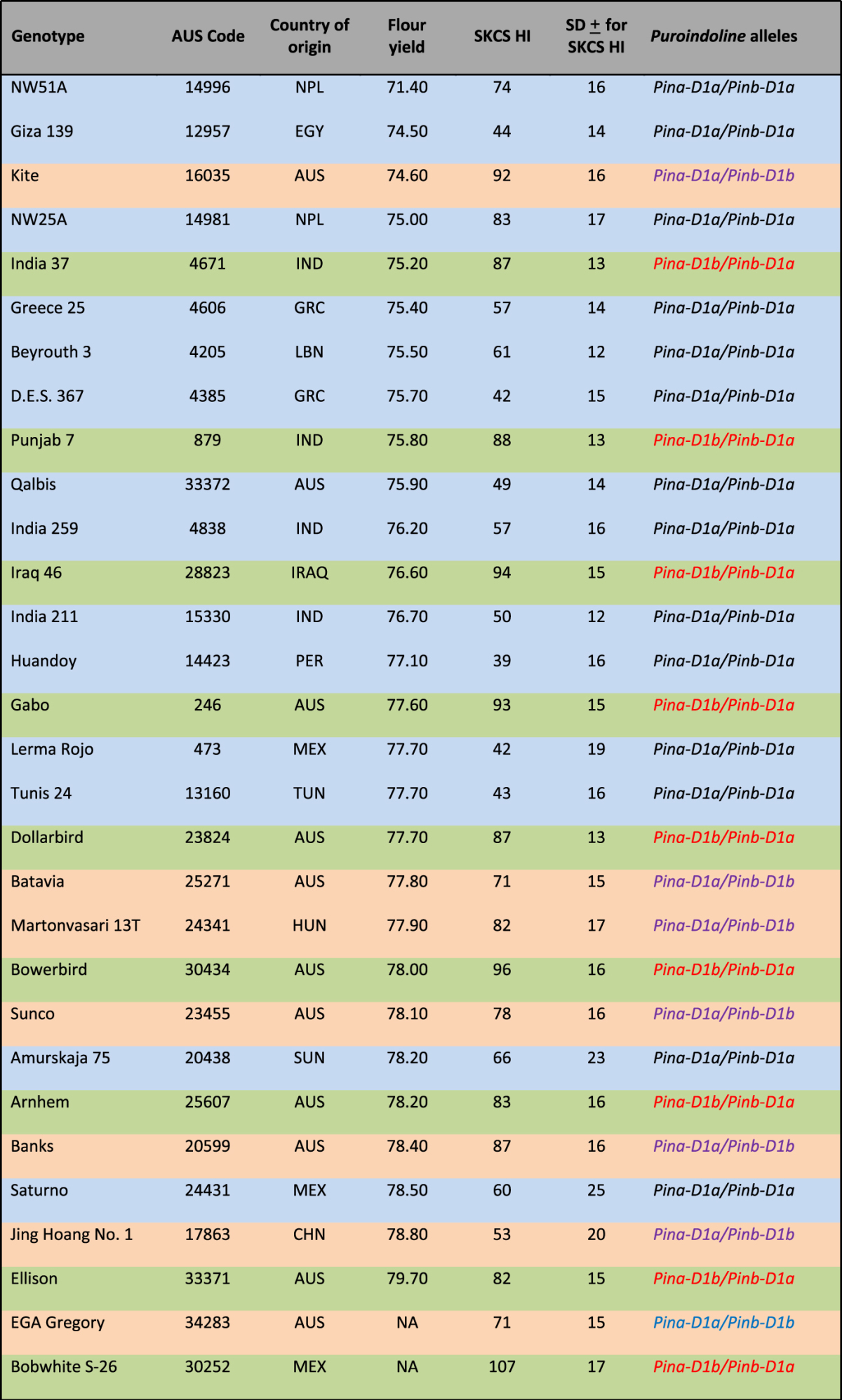
Flour yield of harvested grain, identified by as % of total flour yield, was determined using a Buhler mill. Genotypes were grouped as poor milling (PM) wheats and high milling (HM) wheats if their milling yield was equal to/above or below 77%, respectively. Grain hardness Index (HI) was measured for 300 mature wheat grains using Single Kernel Characterization System (SKCS). SD; standard deviation. Blue colour indicates genotypes with *Pina-D1a/Pinb-D1a*, orange colour indicates genotypes with *Pina-D1a/Pinb-D1b*, and green colour indicates genotypes with *Pina-D1b/Pinb-D1a*.

Wheat Seeds were sourced from the Australian Winter Cereal Collection, Tamworth, Australia.

Among the thirty genotypes, 14 genotypes had *Pina-D1a/Pinb-D1a* alleles of *puroindoline* genes within which 7 genotypes showed soft grain texture (SKCS HI < 50) and 7 showed hard grain texture (SKCS HI > 50), 8 genotypes were hard with *Pina* mutation (*Pina-D1b/Pinb-D1a*), and 7 were hard wheats with *Pinb* mutation (*Pina-D1a/Pinb-D1b)* (Table 1)^18^. Genotypes in HM-group and PM-group were mixture of soft and hard wheats containing different set of *Pin* alleles. All the hard wheats with *Pinb* mutation were good millers, except Giza 139.

### Identification of Differentially expressed genes in good millers using Baggerley’s test

**Figure 1:**
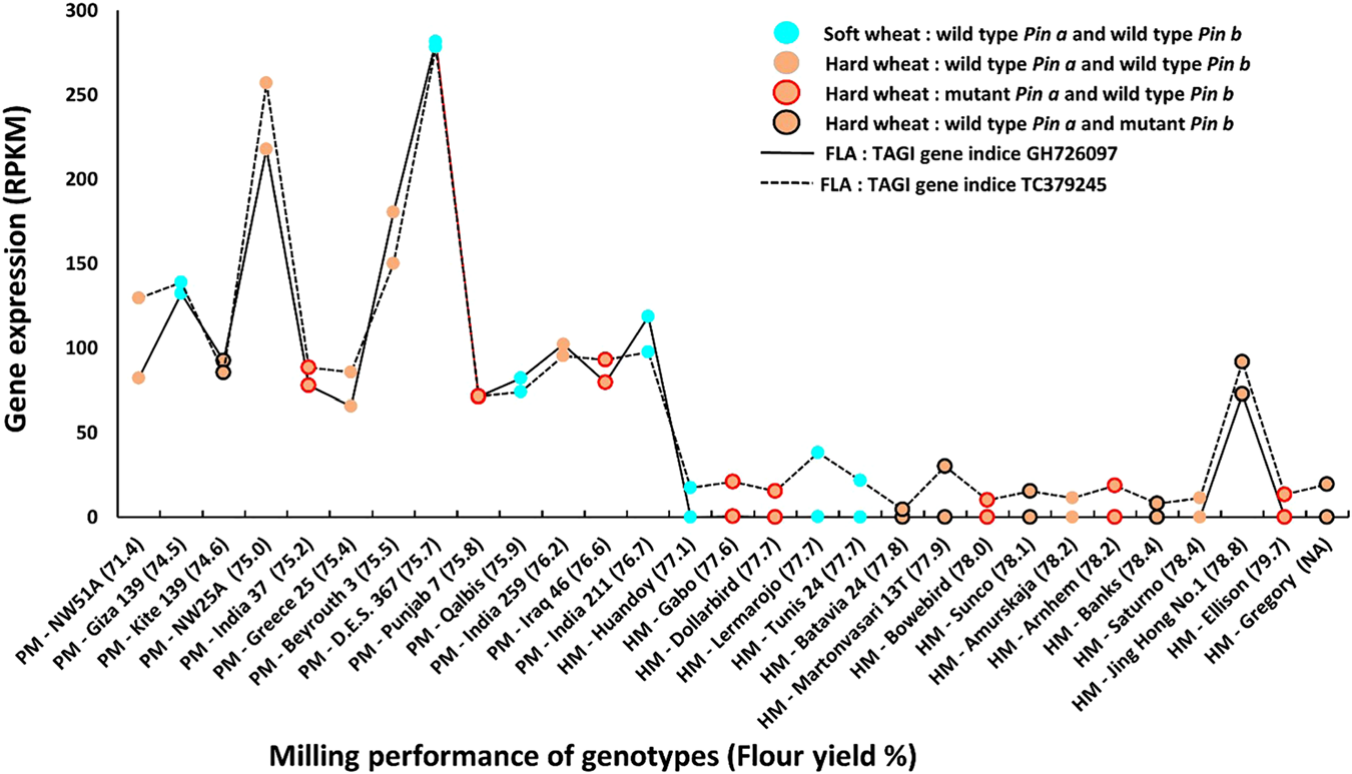
*Fasciclin like arabinogalactan-8* (TAGI; GH726097 and TC379245) expression is down-regulated in developing wheat grains at 14 days post anthesis of wheat genotypes giving a high flour yield. Expression of GH726097 and TC3792456 is measured in reads per kilo base per million mapped reads by analysing Illumina sequencing data on CLC genomic workbench. On the X-axis genotypes are arranged in order of increasing flour extraction rate scores shown within brackets. NA, not analysed; PM, poor milling wheat genotypes giving poor flour yield; HM, high milling wheat genotypes giving high flour yield; *Pin a*, *puroindoline-a* gene allele; *Pin b*, *puroindoline-b* gene allele.

RNA-Seq analysis of genotypes corresponding to the PM-group and HM-group, followed by application of the Baggerley’s test for identification of significant differences led to the identification of twelve genes at 14 DPA and eight genes at 30 DPA that were statistically differentially expressed at a false discovery rate (FDR) p-value of <0.01 (Tables 2 and 3). Sorting the gene list based on proportion difference led to the identification of both up- and down- regulated genes. At 14 DPA, highly significantly differentially expressed transcripts GH726097 and TC379245 showed high homology with a *fasciclin-like arabinogalactan-8* (*FLA-8*). GH726097 and TC379245 were expressed at much lower levels in wheat genotypes with high flour yield; 28.2 and 6.1 fold, respectively (Fig. 1). These two transcripts were also down regulated at 30 DPA in the HM-group. A third transcript (TAGI; TC429180) with homology with *FLA-8* was expressed at higher levels in high milling wheats at 30 DPA. This transcript was 4.5 fold up-regulated in the HM-group. The top two up-regulated gene indices at 14 DPA, TC400069 and TC414662, at a fold change of 20.78 and 10.70 were annotated as Trypsin alpha amylase inhibitor and as wheat Serpin 3 gene, respectively. The top two gene indices at 30 DPA, TC373342 and CJ624986, at a fold change of 9.3 and 45.3 respectively, were annotated as uncharacterized protein from *Triticum urartu* and as 40 s ribosomal protein from *Aegilops tauschii*.

**Table 2:** Differentially expressed genes identified in developing wheat seeds at 14 days post anthesis of wheat genotypes with a high flour yield on milling. Transcriptome data from developing seeds at 14 days post anthesis of 13 poor milling and 19 high milling wheat genotypes obtained by next generation sequencing was subjected to RNA-Seq analysis followed by application of the Baggerley’s test to identify statistically significant differences in gene expression. The list of candidate genes shown are at FDR corrected P value of < 0.01 and sorted based on weighted proportion difference.

Feature ID	Experiment - Fold Change (original values)	Baggerley’s test: Weighted proportions difference	Baggerley’s test: FDR p-value correction	Annotations
GH726097	−28.23	−8.14E-05	2.51E-05	WHEAT Fasciclin FLA8 OS = Triticum aestivum
TC379245	−6.18	−7.33E-05	8.33E-04	WHEAT Fasciclin FLA8 OS = Triticum aestivum
TC388213	−2.62	−1.90E-05	6.71E-04	MAIZE tPR5 OS = Zea mays
CV760049	7.45	2.18E-06	9.59E-03	No BLAST hit
TC434802	3.49	4.06E-06	1.25E-03	TRIUA 78 kDa glucose-regulated OS = Triticum urartu
TC406217	4.42	4.25E-06	0.01	WHEAT Uncharacterized protein OS = Triticum aestivum
TC371705	5.07	5.34E-06	1.37E-04	ARATH polyubiquitin (Fragment) OS = Arabidopsis thaliana
TC433550	27.63	1.68E-05	4.57E-03	70 kDa peptidyl-prolyl isomerase OS = Aegilops tauschii
CJ624986	33.49	3.86E-05	8.59E-04	AEGTA 40 S ribosomal S9–2 OS = Aegilops tauschii
TC419869	7.56	4.11E-05	1.25E-03	AEGTA Cell elongation DIMINUTO OS = Aegilops tauschii
TC414662	10.70	5.96E-05	0.01	WHEAT Serpin 3 OS = Triticum aestivum
TC400069	20.78	1.24E-03	6.71E-04	TRIUA Trypsin alpha-amylase inhibitor CMX1 CMX3 OS = Triticum urartu

**Table 3:** Differentially expressed genes identified in developing wheat seeds at 30 days post anthesis of wheat genotypes with a high flour yield on milling. Transcriptome data from developing seeds at 30 days post anthesis of 13 poor milling and 19 high milling wheat genotypes obtained by next generation sequencing was subjected to RNA-Seq analysis followed by application of the Baggerley’s test to identify statistically significant differences in gene expression. The list of candidate genes shown are at FDR corrected P value of < 0.01 and sorted based on weighted proportion difference.

Feature ID	Experiment - Fold Change (original values)	Baggerley’s test: Weighted proportions difference	Baggerley’s test: FDR p-value correction	Annotations
GH726097	−26.11	−1.94E-05	7.59E-10	WHEAT Fasciclin FLA8 OS = Triticum aestivum
TC379245	−4.55	−1.38E-05	5.47E-04	WHEAT Fasciclin FLA8 OS = Triticum aestivum
TC453372	30.34	3.35E-06	2.67E-03	No BLAST hit
TC416783	2.75	9.91E-06	5.47E-04	HORVD OS = Hordeum vulgare distichum
TC429180	4.53	2.13E-05	5.47E-04	WHEAT Fasciclin FLA8 OS = Triticum aestivum
TC419869	9.21	2.75E-05	4.33E-05	AEGTA Cell elongation DIMINUTO OS = Aegilops tauschii
CJ624986	45.34	3.87E-05	3.72E-04	AEGTA 40 S ribosomal S9–2 OS = Aegilops tauschii
TC373342	9.29	4.08E-05	8.98E-04	TRIUA Uncharacterized protein OS = Triticum urartu

The expression of GH726097 and TC379245 was significantly up-regulated in all of the genotypes of the PM-group but significantly down-regulated in all genotypes from HM-group except for the genotype Jing Hong No.1. Jing Hong No.1 showed very high up-regulation of β-amylase genes (TAGI; CA713114 and CA717472) compared with all other good millers. CA713114 and CA717472 were 707 and 80 fold up-regulated in Jing Hong No.1.

### Characterization of three putative FLA sequences

**Figure 2:**
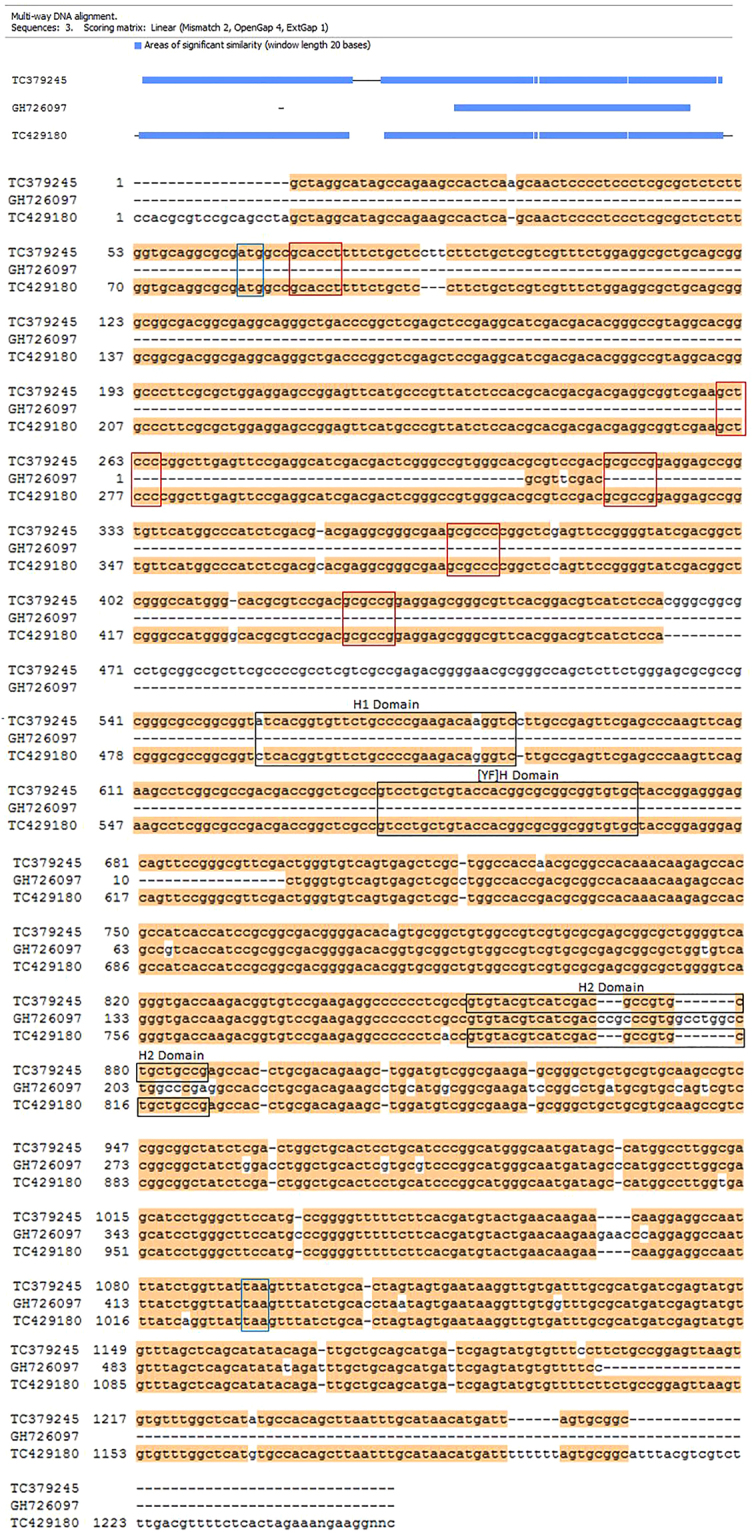
Nucleotide sequence alignments of three *Fasciclin like arabinogalactan* gene sequences corresponding to tentative sequences from the Triticum Aestivum Gene Indices (TAGI), ftp://occams.dfci.harvard.edu/pub/bio/tgi/data/Triticum_aestivum/. Expression of transcripts corresponding to TC379245 and GH726097 is significantly-down regulated at 14DPA, while that of TC429180 is significantly up-regulate at 30 DPA, in high milling wheat genotypes. Red boxes indicate nucleotides encoding for amino acids comprising the AGP Glyco-modules comprising of Alanine and Proline. Black boxes indicate nucleotides encoding for amino acids comprising the FAS domain comprising of H1, [YF]H and H2 conserved regions. Blue Boxes indicate start and stop codons. Global alignment was carried out using Clone Manager (Sci Ed, USA).

The alignment of three differentially expressed putative FLA genes GH726097 (536 bp), TC379245 (1267 bp) and TC429180 (1252 bp) is shown in (Fig. 2). When BLASTn was performed on these three genes using Ensemble with IWGSC survey sequences, two transcripts Traes_2BS_D44E4B43A (cDNA; 1026 bp) and Traes_4BS_03EB5D160 (594 bp) showed the highest similarity. Traes_4BS_03EB5D160 sequence is exactly identical to Traes_2BS_D44E4B43A from base 433 to base 1026. The first of these is located on chromosome 4BS and the later on 2BS. A single FAS domain unique to FLA genes was identified in both these sequences. Limited annotation is available for these genes to date (http://www.wheatgenome.org/). NCBI BLASTn identified DQ872381 as a highly similar gene to GH726097, TC379245 and TC429180. DQ872381 has been annotated as a *fasciclin-like arabinogalactan-8* ^19^.

**Figure 3:**
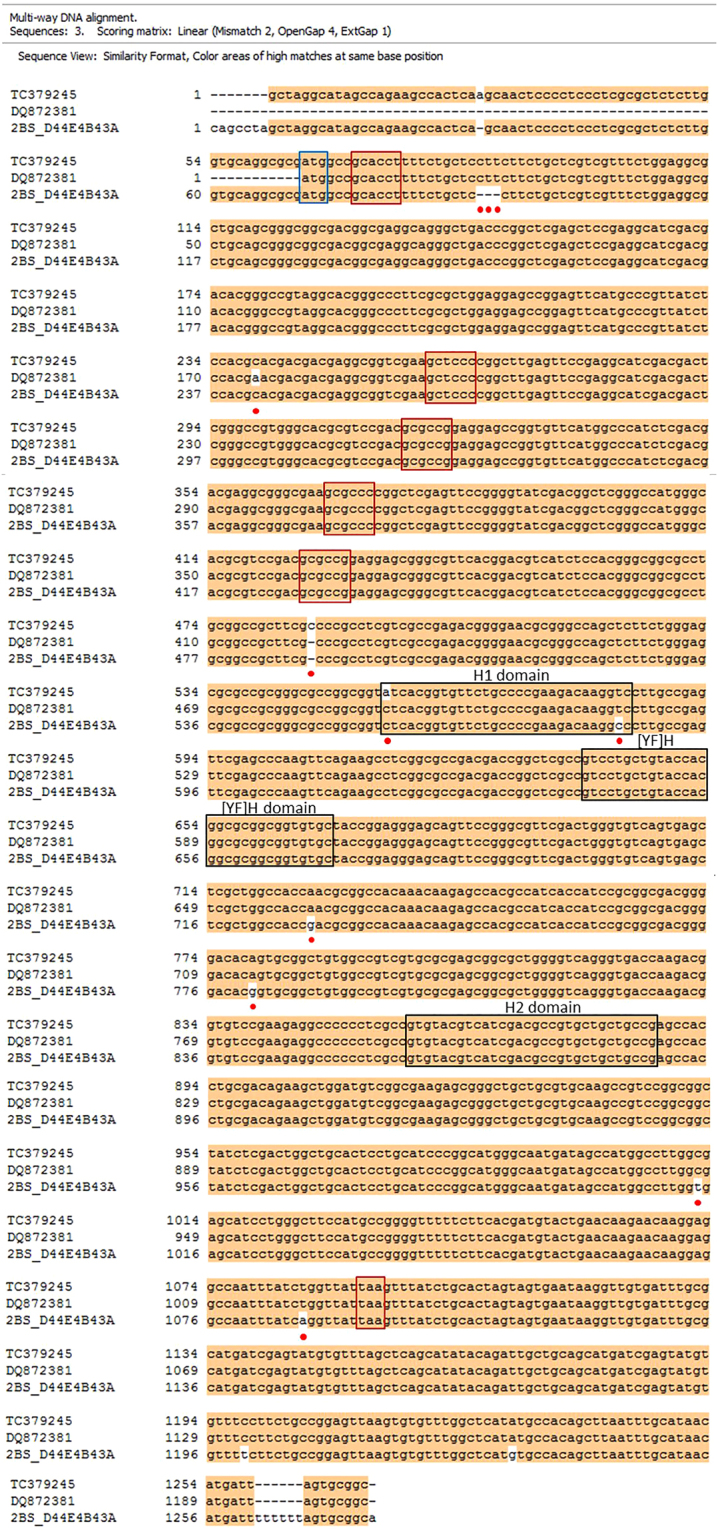
Nucleotide sequence alignments of three *Fasciclin like arabinogalactan* gene sequences TC379245, DQ872381 and, Traes_2BS_D44E4B43A. For TC379245 (Triticum Aestivum Gene Indices), DQ872381 was the top hit on NCBI BLAST and Traes_2BS_D44E4B43A on Ensembl BLAST. Red boxes indicate nucleotides encoding for amino acids comprising the AGP GLyco-modules comprising of Alanine and Proline. Black boxes indicate nucleotides encoding for amino acids comprising the FAS domain comprising of H1, [YF]H and H2 domain. Blue Boxes indicate start and stop codons. Multiway sequence alignment was carried out using Clone Manager (Sci Ed, USA).

**Figure 4:**
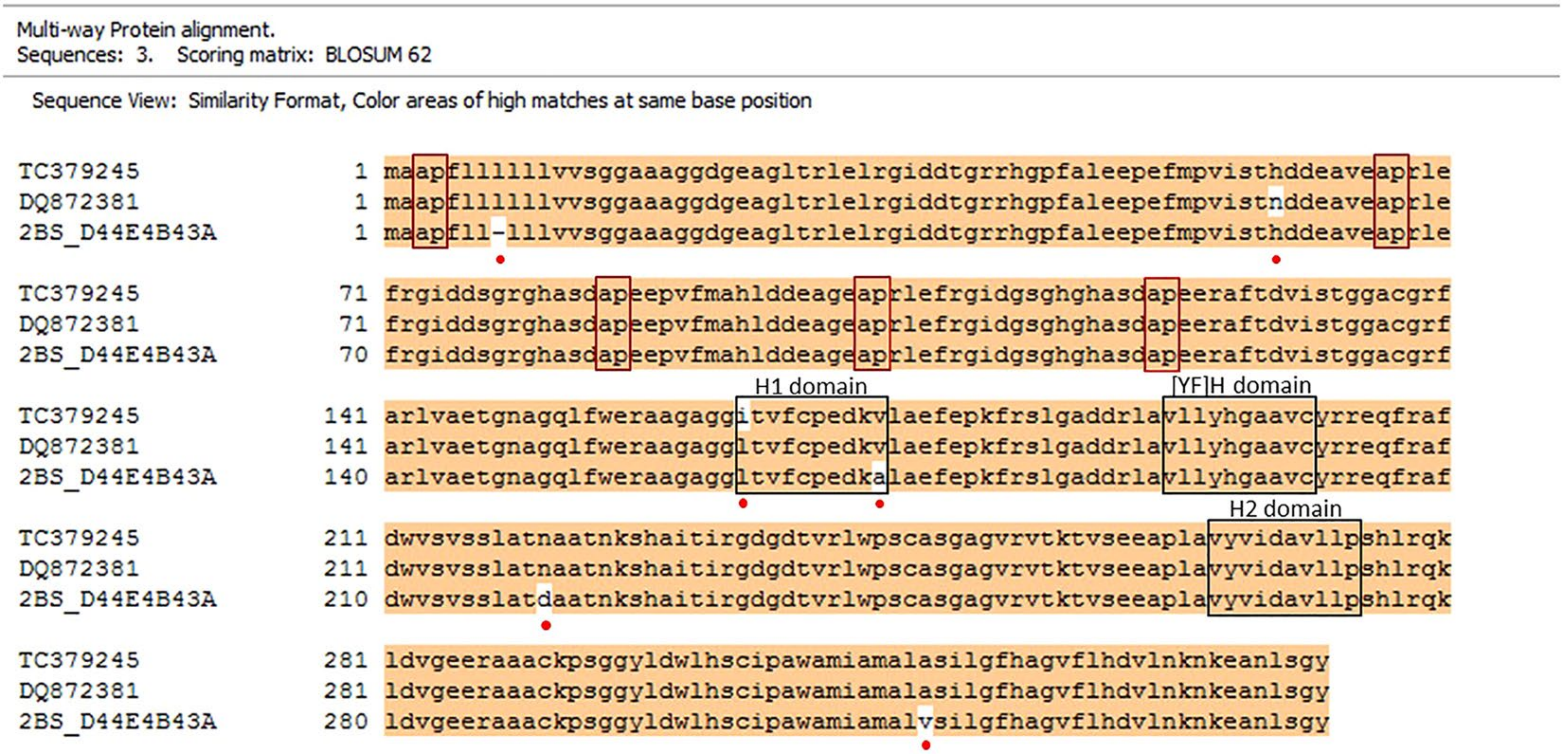
Amino acid sequence alignments of three *Fasciclin like arabinogalactan* polypeptide sequences TC379245, DQ872381 and, Traes_2BS_D44E4B43A. For TC379245 (Triticum Aestivum Gene Indices), DQ872381 was the top hit on NCBI BLAST and Traes_2BS_D44E4B43A on Ensembl BLAST. Red boxes indicate the AGP GLyco-modules comprising of Alanine and Proline. Black boxes indicate amino acids comprising the FAS domain comprising of H1, [YF]H and H2 conserved regions. Red dots indicate conflict positions. Multiway sequence alignment was carried out using Clone Manager (Sci Ed, USA). Note – TC379245 ORF was corrected (‘C’ at position 425 bp removed) before translating the nucleotide sequence.

Nucleotide and the polypeptide sequence alignment performed on Clone Manager (Sci Ed, USA) showed that TC379245 shares 99% sequence identity with Traes_2BS_D44E4B43A and DQ872381 in the coding region (Figs 3 and 4). In TC379245 conserved regions were identified in the FAS domain (Fig. 2), H1:Leu-Thr-Val-Phe-Cys-Pro-Glu-Asp-Lys-Val, [YF]:Val-Leu-Leu-Tyr-His-Gly-Ala-Ala-Val-Cys and H2: Val-Tyr-Val-Ile-Asp-Val-Ile-Ile-Pro. Two notable conflicts were observed in this alignment. First, nucleotide substitution of ‘C’ (cytosine) in the cDNA of TC379245 at position 486 (425 in CDS) was observed to cause a frame shift mutation. However, none of the reads mapped to TC379245 for any of the wheat genotypes showed that substitution. Therefore, the nucleotide ‘C’ at the position 486 was removed from the TC379245 sequence. Second, a deletion of a nucleotide codon was observed in Traes_2BS_D44E4B43A at position between 91^^^92 (21^^^22 in CDS) (corresponding position in TC379245 CDS; 22, 23, 24). This missing codon codes for Leucine at the 7^th^ position in the polypeptide chain of DQ872381 and Traes_2BS_D44E4B43A. All the nucleotide conflicts and the corresponding changes in the amino acid polypeptide are shown in Table 4.

**Table 4:** Nucleotide conflicts and corresponding changes in amino acid polypeptide are shown in table. Nucleotide and translated amino acid sequences of TC379245, DQ872381 and Traes_2BS_D44E4B43A were aligned in Clone manager to find out sequence similarity. Note – Nucleotide at position 486 bp (422 bp in CDS) in TC379245 was removed before translation as it causes frameshift mutation.

Nucleotide base position in cDNA of TC379245 (in CDS)	Nucleotide in TC379245 (amino acid change and position in polypeptide)	Nucleotide in DQ872381 (amino acid change)	Nucleotide in Traes_2BS_D44E4B43A (amino acid change)	Nucleotide in TC379245 consensus^*^
86, 87, 88 (22, 23, 24)	CTT (Leu-7)	CTT	—	CTT
239 (175)	C (His-59)	A (Asn)	C	A
486 (422)	C	—	—	—
555 (491)	A (Ile-164)	C (Leu)	C	C
583 (519)	T (Val-173)	T	C (Ala)	T
726 (662)	A (Asn-221)	A	G (Asp)	A
779 (715)	A (Tyr-238)	A	G (Tyr)	A
1012 (948)	C (Ala-316)	C	T (Tyr)	C
1085 (1021)	T (Ser-340)	T	A (Ser)	T

^*^TC379245 consensus was obtained from reads mapped to the reference sequence of TC379245.

The TC429180 nucleotide sequence showed 87% identity with TC379245. Deletion of a 78 bp sequence was observed in TC429180, from position 461 to 541, corresponding to TC379245. TC429180 RNA-seq mapping files from all the wheat genotypes were manually checked to determine coverage at the conflict region. In none of the files were reads observed to span the region of conflict. In none of the hits obtained for TC429180 on NCBI and Ensemble BLAST was the 78 bp deletion observed. Therefore, true identity of this deletion was not confirmed. A FAS domain was identified in the nucleotide sequence of TC429180 (Fig. 2). The translated amino acid sequence shows 99% sequence similarity to the TC379245 polypeptide before the start of deletion site but the later part of the sequence shows no similarity due to the apparent frameshift in the TC429180 polypeptide. However, as the 78 bp deletion couldn’t be confirmed the true sequence of the TC429180 polypeptide couldn’t be identified.

GH726097 showed 90% sequence similarity to the TC379245 in the aligned region (Fig. 2). The GH726097 sequence is less than half the length of TC379245 and no open reading frame was identified within the sequence and thus it is likely to be an incomplete transcript sequence. No FAS domain was identified in this nucleotide sequence. Reads mapped to these two transcripts were sequence specific/unique which provides the evidence for them to be two different genes.

**Figure 5:**
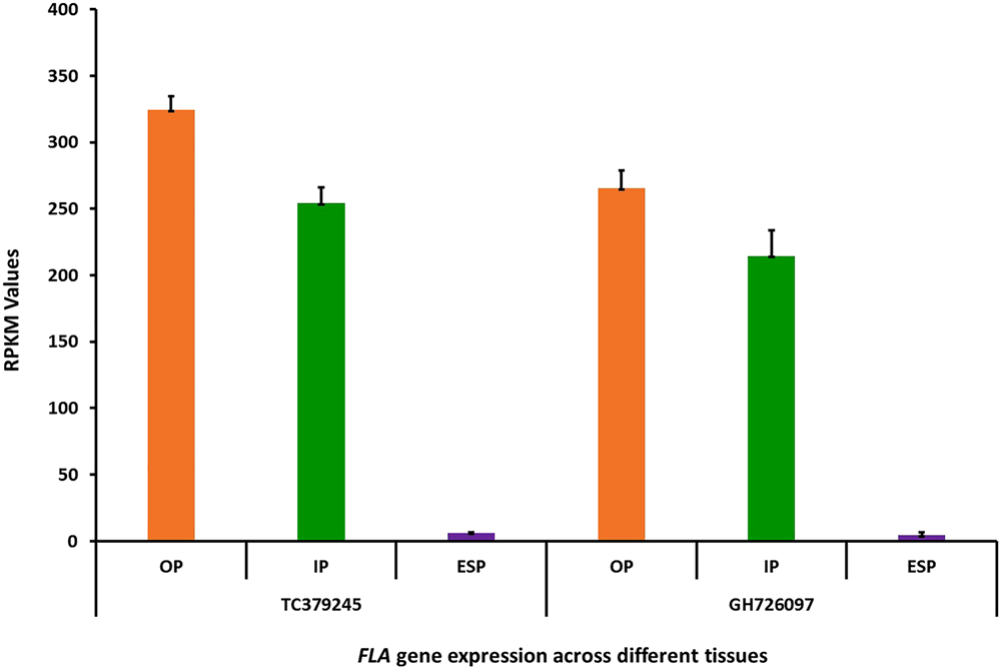
Tissue-specific expression (RPKM) of *FLA* transcripts in developing wheat seeds. The mean of three biological replicates^20^ is presented for different tissues of the developing wheat grain at 12 days-post-anthesis in the cultivar, Holdfast. **ESP:** Endosperm; **IP:** inner pericarp; **OP:** outer pericarp; **TC378245 and GH726097**: transcript IDs correspond to *FLA* genes from the TAGI database Raw reads obtained from data reported by Pearce et al. (2015)^20^ (http://www.ebi.ac.uk/arrayexpress/experiments/E-MTAB-3103/samples/) were processed by RNA-Seq analysis to deduce *FLA* expression, using CLCbio Workbench (Qiagen Bioinformatics, Denmark).

Analysis of published wheat transcriptome data was used to examine the tissue specificity of expression of these genes. In the grain, both transcripts have been reported in pericarp (inner and outer) but not in endosperm tissue (Fig. 5, transcriptome data from^20^).

## Discussion

Variation in the gene pool provides the resource for breeding improved genotypes^17^. The relationship between gene expression and flour yield in milling was analysed for 30 diverse wheat genotypes. Gene expression varies throughout seed development^21^ with genes expressed at different stages contributing to the final composition and properties of the grain. Much of the variation in hardness of the wheat genotypes was explained by differences in *Pin* genes^18^ and these loci have been associated with flour yield variation in some germplasm^8^ but hardness did not explain most of the variation in milling performance (Fig. 1).

Down regulation of expression of transcripts (TAGI; TC379245 and GH726097) encoding cell adhesion proteins, *FLA-8*, was found at the critical mid grain development stage (14 DPA) and also at a later stage, close to maturity (30 DPA) in genotypes that gave high flour yield (Table 2). Reduced expression of these genes may be associated with a reduction in the structural strength of the grain. These changes are likely to result in a grain that breaks up more easily in the mill and yields greater quantities of flour.

The arabinogalactan proteins (AGPs) are highly glycosylated proteins that are rich in hydroxyproline and found in plant cell wall and plasma membrane^22^. AGPs that contain putative cell adhesion domain known as fasciclin domain are known as fasciclin like arabinogalactan proteins. FLA has been implicated in cell adhesion and may link the cell membrane and cell wall. AGPs carry glycosylphosphotidylinositol (GPI) anchor at the C-terminus which signals the peptide to the plasma membrane. However, wheat *FLA-8* has a shorter GPI-anchor which raises a question about its binding to plasma membrane^19^. But it might have a role in cell to cell and cell to extracellular environment interaction as suggested by Faik *et al*.^19^. Modification of a *FLA* in poplar^23^ has been shown to reduce the mechanical strength of plant tissues^24,25^. Human selection for higher flour yield has apparently resulted in the selection of wheat genotypes with greatly reduced levels of expression of this gene. Nanospherical arabinoxylan proteins have been recently identified as the adhesive component secreted by the climbing plant, English ivy^26^.

A third transcript (TAGI; TC429180) with homology with *FLA-8* was expressed at higher levels in high milling wheats at 30 DPA. The tissue specificity of expression is not known in this system but varies significantly in other plants^25^. Most gene expression at 30 DPA may be in the embryo as the endosperm is terminally differentiated by this stage.

Recently Wilkinson *et al*.^27^ (2017) reported that the product of the granule softness protein gene was an AGP. This would complicate any attempts to relate FLA levels to milling yield. Methods for distinguishing the FLAs from other AGPs would be required. Hard wheats are generally considered to be better milling wheats^14^ and a reduced expression of AGP encoded by the *Gsp-1* gene at the hardness locus might contribute to improved flour yield. However this study showed that flour yield was not associated with the *pin* genotype at the hardness locus (Fig. 1).

*FLA-8* related genes were found to be located on chromosome 2BS and 4BS in the Chinese Spring wheat genome assembly with limited annotation to date (http://www.wheatgenome.org/). A major QTL for flour yield has been reported from chromosome 2B^7,12^. The QTL on chromosome 2BS had the highest overall LOD score (13.1) in analysis of the progeny of a Sunco X Tasman cross, explaining 14.4–31.3% of the variation in flour yield at different sites^7^. Sunco was shown to have low expression of the *FLA* gene in the current study and was the source of the high flour yield in the Sunco X Tasman cross^7^. A QTL on chromosome 4B was the second most significant flour yield QTL in this cross. However in another cross, Katepwa X CD87, the 4B QTL was the most important and explained 13.6–23.8% of variation in flour yield. The coincidence of the structural genes encoding FLA and the chromosomal locations of the major know QTLs for milling yield in these crosses on chromosomes 4B and 2 A provides strong confirmation of the significance of these associations.

The strength of the endosperm may be influenced by properties of the protein bodies and starch granules^28^ that fill the endosperm cells and account for most of the grain content. The expression of storage protein genes was significantly altered in wheat with high flour yield. Several genes may contribute to changes in protein synthesis in the better milling wheat genotypes. The expression of 40 S ribosomal protein was significantly higher in the better milling wheat genotypes at both 14 and 30 DPA (Tables 2 and 3). This protein may regulate protein synthesis and down regulation of this gene has been reported in a late ripening citrus genotype^29^. Selection for protein composition that better suits end uses such as bread making may have been more intense in the development of genotypes also selected for milling quality.

Earlier studies have suggested that starch granule size distribution may be associated with flour yield differences^9,14^. This suggests that modification of starch may contribute to flour yield. Jing Hoang No.1 had a very high level of expression of β-amylase and was the only genotype giving high flour extraction without significantly reduced expression of the *FLA-8* genes. High flour yield in wheat may be the result of unintended human selection for different types of genetic variation in different genotypes. New deliberate combinations of these individual genes may have application in development of wheat genotypes with very high flour yields. Other wheat is processed by directly grinding the grain. This type of flour is used to make products such as chapatti. Wheat genotypes in regions where these products predominate may not have been subjected to selection for flour yield in roller milling.

The tissue specificity^30^ of the genes identified in this study may be important as changes in the expression of many of these genes may have been achieved by human selection for flour yield without perturbing critical gene expression in other parts of the plant. These genes are specifically expressed in the pericarp where they may influence the adherence of the pericarp to the Aleurone/starchy endosperm. In hexaploid wheat modification of sub-genome specific expression (in the A, B or D sub-genomes) may allow tissue specific alteration of function. Recent analysis of expression of defence genes and response to pathogen infection has been shown to result in highly sub-genome specific expression^31^.

Analysis of the wheat grain transcriptome has identified a gene controlling bread quality^32^ and new pathways for carbon assimilation^33^. Although the gene controlling bread quality has at least five copies in the wheat genome with high sequence homology, the cause of the differential expression of this gene was identified to be the promoter region of a specific allele of this gene. A simple PCR-test was develop which allowed the identification of wheat genotypes for the presence or absence of the specific allele of gene controlling bread quality^32^ which has been used by CIMMYT^34^. A similar strategy can be used to identify the presence or absence of the specific FLA allele which corresponds to high FLA-8 expression and thereby select for wheat genotypes for good milling.

Selection for these genes and the flour yield genes identified in the present study provides a new set of tools for wheat breeders that should reduce the constraints of selection for wheat quality^35^ on the rate of genetic gain in wheat breeding and assist the selection of new genotypes to support the adaptation of wheat production to climate change^36,37^.

## Experimental Procedures

### Plant Material

Wheat grains of a worldwide set of wheat genotypes were sourced from Australian Winter Cereal Collection (AWCC), Tamworth, NSW, Australia; now known as Australian Grains Genebank (AGG), Horsham, Victoria, Australia (http://www.seedpartnership.org.au/associates/agg). Plants of all thirty wheat genotypes were grown under field conditions at Narrabri (NSW, Australia) and harvested at maturity. Another trial was grown at Biloela (Qld, Australia). The conditions at this site were harsher resulting in some varieties not performing well. Milling of these samples gave flour yield data that showed a highly significant (P = 0.01) correlation with that from the Narrabri site but the data was not included in the association analysis because it was considered a far less reliable measure of the flour milling qualities of these genotypes when grown in unfavourable environments.

### Test milling and grain hardness

Milling yield of harvested grain, identified by as % of total flour yield, was determined using a Buhler mill as explained by Edwards *et al*.^9^. Wheat genotypes were grouped as poor milling (PM) wheats and high milling (HM) wheats if their milling yield was equal to/above or below 77%, respectively.

Grain hardness for all the genotypes was measured using Single Kernel Characterization System (SKCS) as described by Nirmal *et al*.^18^. Puroindoline alleles in all the genotypes were also characterized as described in Nirmal *et al*.^18^.

### RNA isolation, cDNA preparation and NGS sequencing

Total RNA was isolated from the whole caryopsis at 14 days post anthesis (DPA) and at 30 dpa^38^. cDNA was prepared and used to produce indexed Illumina NGS libraries which were then multiplexed to allow the sequencing of eight indexed libraries in one lane on a GA IIx Illumina sequencing platform to obtain 100 bp paired-end reads. RNA sequencing of the thirty wheat genotypes generated a total of ~2.5 million to ~7.2 million reads varying with the genotype.

### RNA-Seq analysis

All NGS data was analysed using CLC Genomic Workbench Version 9.0 (CLC Bio, Qiagen). All the reads were trimmed for quality to exclude calls with Fred quality scores less than 20. Following trimming all sequence data was subjected to quality analysis. After quality check reads were subjected to RNA-seq analysis. Triticum aestivum gene index (TAGI) database (ftp://occams.dfci.harvard.edu/pub/bio/tgi/data/Triticum_aestivum/) which contains 221,925 tentative consensus sequences obtained from EST, cDNA and mRNA libraries was used as a reference database for RNA-seq analysis. Read alignment parameters length fraction and similarity fraction were set to 0.8 and 0.9, respectively. Paired reads were counted as two. Gene expression was normalised by calculating reads per kilo base per million mapped reads (RPKM).

Tissue specificity of expression of *FLA* genes in wheat grain was investigated in transcriptome data available from published data^20^ for outer pericarp, inner pericarp, and endosperm tissues at 12 days-post-anthesis in developing wheat grains (cv. Holdfast) by RNA-Seq analyses using the TAGI reference in CLC genomics workbench. The data included three biological replicates and since there is was no significant difference between the replicates the mean values are shown in Fig. 5.

### Identification of differentially expressed genes

RNA-seq data of the PM-group and the HM-group was subjected to proportion-based Baggerley’s test^39^ within CLC workbench to identify differentially expressed genes (DEGs) in the HM-group when compared to the PM-group. Differentially expressed genes with FDR (false discovery rate) p-value of < 0.01 were considered as statistically significant candidate genes associated. A further sorting of the DEGs was undertaken based on Baggerley’s weighted proportion difference, to identify those genes with consistent expression difference in genotypes within the PM and HM wheat genotypes.

### Functional annotations

Differentially expressed genes were functionally annotated using Blast2GO PRO^40^.

### Data Availability Statement

All NGS sequence data as raw data was submitted to NCBI at the Sequence Read Archive (SRA) and is available as SRA Submission # SUB2954840 (under BioProject# PRJNA392390 and BioSample #SUB2843912).
